# Cytosolic DNA sensors activation of human astrocytes inhibits herpes simplex virus through IRF1 induction

**DOI:** 10.3389/fcimb.2024.1383811

**Published:** 2024-05-14

**Authors:** Yu Liu, Xi-Qiu Xu, Wei-Jing Li, Biao Zhang, Feng-Zhen Meng, Xu Wang, Safah M. Majid, Zihan Guo, Wen-Zhe Ho

**Affiliations:** ^1^ Department of Pathology and Laboratory Medicine, Temple University Lewis Katz School of Medicine, Philadelphia, PA, United States; ^2^ College of Life Sciences and Health, Institute of Biology and Medicine, Wuhan University of Science and Technology, Wuhan, Hubei, China; ^3^ School of Basic Medical Sciences, Wuhan University, Wuhan, Hubei, China

**Keywords:** astrocytes, interferon regulator factor 1 (IRF1), dsDNA, interferons (IFNs), IFN-stimulated genes (ISGs)

## Abstract

**Introduction:**

While astrocytes participate in the CNS innate immunity against herpes simplex virus type 1 (HSV-1) infection, they are the major target for the virus. Therefore, it is of importance to understand the interplay between the astrocyte-mediated immunity and HSV-1 infection.

**Methods:**

Both primary human astrocytes and the astrocyte line (U373) were used in this study. RT-qPCR and Western blot assay were used to measure IFNs, the antiviral IFN-stimulated genes (ISGs), IFN regulatory factors (IRFs) and HSV-1 DNA. IRF1 knockout or knockdown was performed with CRISPR/Cas9 and siRNA transfection techniques.

**Results:**

Poly(dA:dT) could inhibit HSV-1 replication and induce IFN-β/IFN-λs production in human astrocytes. Poly(dA:dT) treatment of astrocytes also induced the expression of the antiviral ISGs (Viperin, ISG56 and MxA). Among IRFs members examined, poly(dA:dT) selectively unregulated IRF1 and IRF9, particularly IRF1 in human astrocytes. The inductive effects of poly(dA:dT) on IFNs and ISGs were diminished in the IRF1 knockout cells. In addition, IRF1 knockout attenuated poly(dA:dT)-mediated HSV-1 inhibition in the cells.

**Conclusion:**

The DNA sensors activation induces astrocyte intracellular innate immunity against HSV-1. Therefore, targeting the DNA sensors has potential for immune activation-based HSV-1 therapy.

## 1 Introduction

Astrocytes participate in the CNS innate immunity against invasive pathogens (Sofroniew, 2020), including HSV-1 (Li et al., 2012; Xu et al., 2023). Studies have shown that astrocytes are directly involved in HSV-1 infection of the CNS, as they are a major target for the virus (Reinert et al., 2012; Yue et al., 2013). While microglia are commonly viewed as the primary innate immune cells in the brain, increasing evidence indicates that astrocytes also participate in the local immune response triggered by inflammation, viral infections, and pathological brain injury repair (Farina et al., 2007). Like microglia, astrocytes are equipped with a variety of pattern recognition receptors (PRRs) such as NOD-like receptors (NLRs), RIG-I-like receptors (RLRs), Toll Like Receptors (TLR), and the DNA sensors. These sensors have important roles in host intrinsic/innate immunity against invading pathogens including both RNA and DNA viruses. Through recognizing conserved determinants of viral origin, these receptors detect either viral RNA or DNA and activate the antiviral immune responses, producing IFNs and IFN-stimulated genes (ISGs) (Acchioni et al., 2015; Wei et al., 2016; Hao et al., 2020; Ren et al., 2023). IFNs and ISGs as well as other cellular factors are the key components of the antiviral innate immunity against HSV-1 infection (Mossman and Ashkar, 2005; Chew et al., 2009; Knipe, 2015). Therefore, these virus detection receptors are critical for preventing and controlling viral infection/replication.

There is limited information about whether the DNA sensor activation can inhibit HSV-1 infection of human astrocytes and the mechanisms associated with. Studies have shown that the DNA sensors can recognize several DNA viruses, including HSV-1 (Howard et al., 2021; Sui et al., 2021; Howard et al., 2022). Importantly, the DNA sensor activation by viral DNAs results in activation of IFN signaling pathway and induction of antiviral factors. To date, multiple DNA sensors have been identified to detect intracellular viral DNA and elicit the innate immune responses (Unterholzner et al., 2010; Zhang B. et al., 2023). The DNA sensor IFI16 could recognize the encapsulated HSV DNA and activate IFN regulator factor 3 (IRF3)-mediated signaling pathway (Unterholzner et al., 2010; Orzalli et al., 2012). In addition, as a key cytosolic dsDNA sensor, cGAS could interfere with multiple HSV-1 gene products and induces IFN production (Reinert et al., 2016). Guo et al. revealed that ZBP1/DAI triggers necroptosis against HSV-1 by the host innate immune system, and this process does not require IFN signaling for the initiation of necroptosis (Guo et al., 2018). Sui et al. showed that the DNA sensor Ku70 activation could induce the type III IFNs through the activation of IRF1, IRF3 and IRF7 when cells were exposed to cytosolic DNA or infected with HSV-2 (Sui et al., 2017). In the present study, we examined whether activating DNA sensors of human astrocytes by a dsDNA analog, poly(dA:dT), can induce IFN-driven intracellular immunity and inhibit HSV-1 infection/replication. We also investigated the mechanisms of poly(dA:dT)-elicited IFN signaling pathway activation and HSV-1 inhibition in human astrocytes.

## 2 Materials and methods

### 2.1 Cell lines and virus

Human primary astrocytes were obtained from Comprehensive NeuroAIDS Center at the Temple University Lewis Katz School of Medicine. The cells were cultured in the DMM/F-12 medium contained with 15% FBS, 50μg/ml Gentamicin, 5μg/ml Fungizone, 10μg/ml Insulin. Human malignant glioma cell line (U373) was purchased from ATCC. The cells were cultured in DMEM medium containing 10% FBS, 1×Penicillin-Streptomycin (PS), 1×HEPES. A highly neurovirulent HSV-1 17syn^+^ strain was kindly provided by Dr. James Lokensgard (University of Minnesota Medical School). HSV-1 17syn^+^ was propagated in rabbit skin fibroblasts (CCL68; ATCC) at a multiplicity of infection (MOI) of 0.01.

### 2.2 Reagents

Transfection reagent LyoVec™, synthetic dsDNA analog poly(dA:dT) were purchased from Invivogen (San Diego, CA, USA). Antibodies against GAPDH, ZBP1, STING, TLR3, RIG-I, MDA5, MxA, ISG56, Viperin, IRF3, phospho-IRF3, IRF7, STAT1, phospho-STAT1 were obtained from Cell Signaling Technology (Danvers, MA, USA). Antibodies against IFI16, IRF1 and IRF9 were obtained from Santa Cruz (Dallas, TX, USA). Antibodies against HSV gD and gB were obtained from Abcam. LentiCRISPRV2, pMD2.G and psPAX2 plasmids were kindly offered by Dr. Jian Huang at the Temple University Lewis Katz School of Medicine.

### 2.3 RNA extraction and quantification

Total Cellular RNAs were extracted from cells with Tri-reagent (Molecular Research Center, Cincinnati, OH, USA), according to the manufacturer’s instructions. RNAs were reverse transcribed using the random primer, dNTPs, M-MLV reverse transcriptase and RNase inhibitor (Promega Co., Madison, WI, USA). Real-time PCR was performed with SYBR GREEN PCR master mix (Bio-Rad Laboratories, Hercules, CA, USA). GAPDH mRNA was used as an endogenous reference to normalize the quantities of the target mRNA. The sequences of the oligonucleotide primers are shown in **Table 1**.

**Table 1:** Primer pairs for the real-time PCR.

Gene	Sequence	
	Forward (5’-3’)	Reverse (5’-3’)
GAPDH	GGTGGTCTCCTCTGACTTCAACA	GTTGCTGTAGCCAAATTCGTTGT
IFN-α	TTTCTCCTGCCTGAAGAACAG	GCTCATGATTTCTGCTCTGACA
IFN-β	GCCGCATTGACCATCTATGAGA	GAGATCTTCAGTTTCGGAGGTAAC
IFN-λ1	CTTCCAAGCCCACCCCAACT	GGCCTCCAGGACCTTCAGC
IFN-λ2/3	TTTAAGAGGGCCAAAGATGC	TGGGCTGAGGCTGGATACAG
IRF1	TGAAGCTACAACAGATGAGG	AGTAGGTACCCCTTCCCATC
IRF3	ACCAGCCGTGGACCAAGAG	TACCAAGGCCCTGAGGCAC
IRF5	AAGCCGATCCGGCCAA	GGAAGTCCCGGCTCTTGTTAA
IRF7	TGGTCCTGGTGAAGCTGGAA	GATGTCGTCATAGAGGCTGTTGG
IRF9	GCATCAGGCAGGGCACGCTGCACC	GCCTGCATGTTTCCAGGGAATCCG
Viperin	TGGGTGCTTACACCTGCTG	TGAAGTGATAGTTGACGCTGGT
ISG56	TTCGGAGAAAGGCATTAGA	TCCAGGGCTTCATTCATAT
MxA	GCCGGCTGTGGATATGCTA	TTTATCGAAACATCTGTGAAAGCAA
HSV gD	ATCCGAACGCAGCCCCGC	TCTCCGTCCAGTCGTTTAT

### 2.4 Western blot assay

Total cell lysates of human astrocytes were prepared by using RIPA buffer (SIGMA, MO, USA) with 1% protease inhibitor and 1% phosphatase inhibitor (SIGMA, MO, USA). Equal amounts of protein lysates (20μg) were separated on 4% to 12% sodium dodecyl sulfate (SDS) polyacrylamide gel electrophoresis precast gels and transferred to the polyvinylidene difluoride membrane (Millipore, Eschborn, Germany). The blots were incubated with primary antibodies in 2% nonfat milk in phosphate-buffered saline with 0.05% Tween 20 (PBST) overnight at 4°C. The blots were then washed with PBST and further incubated with horseradish peroxidase-conjugated appropriate second antibodies in 2% nonfat milk PBST for one hour at room temperature. Blots were developed with enhanced chemiluminescence (Amersham, Bucks, UK) in a Fuji Film LAS-4000 imaging analyzer (GE Life Sciences, NJ, USA).

### 2.5 siRNA transfection

Control siRNA, IRF1 siRNA (Cat#: MHSXX0020) and DharmaFECT transfection reagent were obtained from Dharmacon (CO, USA). 30nM siRNA was transfected into primary human astrocytes and U373 cells according to the manufacturer’s instructions. The cellular RNA or protein were extracted at 24h or 48h post-transfection, respectively.

### 2.6 Generation of IRF1^-/-^ U373 cells

U373 cells lacking IRF1 were generated using CRISPR/Cas9 technology. Briefly, LentiCRISPRV2 plasmids were digested and ligated with annealed guide RNAs, then transfected with pMD2.G and psPAX2 into 293T cells using FuGENE^®^ 6 transfection reagent (Promega, USA). The supernatant was collected after 48h and 72h, then was centrifuged at 3000rpm for 15min. U373 cells were infected with lentivirus supernatant and selected with puromycin (2μg/ml), the IRF1 protein level was determined by Western blot assay. The oligo sequences for IRF1 guide RNAs are shown in **Table 2**.

**Table 2:** The oligo sequences of IRF1 guide RNAs.

	Forward (5’-3’)	Reverse (5’-3’)
IRF1 gRNA1	**CACCG**CTCATGCGCATCCGAGTGAT	**AAAC**ATCACTCGGATGCGCATGAG**C**
IRF1 gRNA2	**CACCG**TCTCATGCGCATCCGAGTGA	**AAAC**TCACTCGGATGCGCATGAGA**C**
IRF1 gRNA3	**CACCG**ATGCCTGTTTGTTCCGGAGC	**AAAC**GCTCCGGAACAAACAGGCAT**C**

### 2.7 ELISA assay

IFN-β and IFN-λ1 protein levels in the human primary astrocytes or U373 cells culture supernatant were examined by ELISA (Invivogen, USA). Assays were performed as instructed by the manufacturer.

### 2.8 Data analysis

Data were shown as the mean ± standard deviation (mean ± SD) and analyzed by Student’s t-test. Calculations were performed with GraphPad Prism Statistical Software (GraphPad Software Inc., San Diego, CA, USA). Statistical significance was defined as *p < 0.05 or **p < 0.01.

## 3 Results

### 3.1 Poly(dA:dT) inhibits HSV-1 DNA replication of human astrocytes

**Figure 1:**
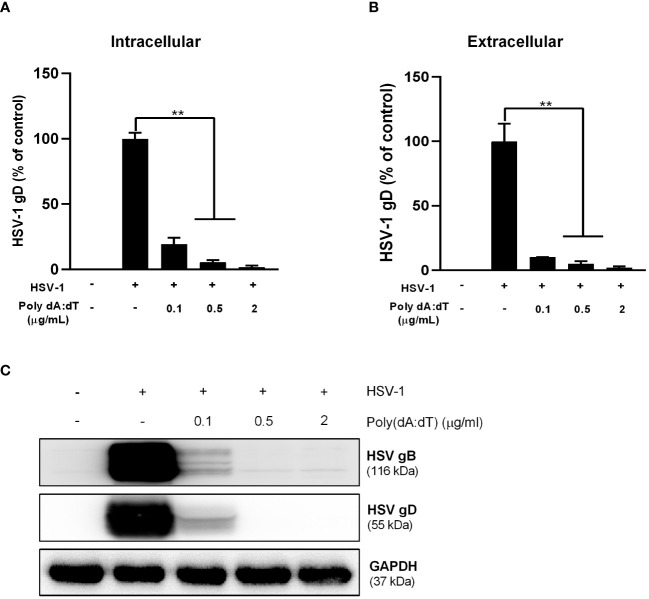
Poly(dA:dT) inhibits HSV-1 DNA replication. U373 cells were pretreated with poly(dA:dT) for 24h and followed by HSV-1 (MOI of 0.02) infection for 2h. The cells were then washed with PBS. Twenty-four hours post infection (PI), intracellular **(A)** and extracellular **(B)** DNA were collected and analyzed by the real-time PCR for HSV-1 gD expression. **(C)** Total cellular proteins were extracted and analyzed by Western blot for HSV-1 gD and gB protein expression. Data shown in **(A, B)** were the mean ± SD of three independent experiments with triplicate wells (**P < 0.01).

We first examined the effect of poly(dA:dT) on HSV-1 infection of human astrocytes. U373 cells were pretreated with poly(dA:dT) for 24h prior to HSV-1 infection. As demonstrated in **Figures 1A, B**, poly(dA:dT)-treated cells had much lower levels of intracellular and extracellular HSV-1 gD DNA than untreated cells. In addition, we observed that total intracellular HSV-1 gB and gD protein levels in the treated cells were significantly lower than those in the control cells (**Figure 1C**). The inhibitory effects of poly(dA:dT) on HSV-1 were dose-dependent (**Figure 1**).

### 3.2 Poly(dA:dT) induces DNA and RNA sensors

**Figure 2:**
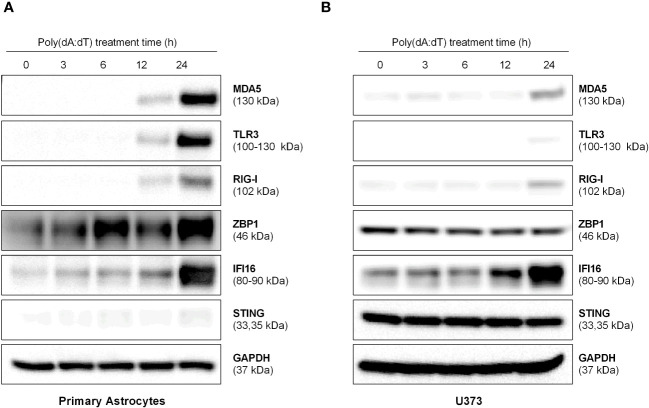
Poly(dA:dT) induces IFI16 and RNA sensors. The primary human astrocytes **(A)** and U373 cells **(B)** were transfected with 0.5μg/ml of poly(dA:dT) at the indicated times. The cellular proteins were extracted for the indicated sensors. Data shown are representative of three independent Western blot experiments.

Because many cellular pattern recognition receptors are involved in sensing cytosolic DNA, we examined the effect of poly(dA:dT) on several DNA and RNA sensors expression in human astrocytes. As show in **Figures 2A, B**, poly(dA:dT) treatment induced the expression of the three key RNA sensors (MDA5, TLR3 and RIG-I) in both primary human astrocytes and U373 cells. Poly(dA:dT)-treated cells also showed higher expression of IFI16 and ZBP1 in primary human astrocytes (**Figure 2A**), although only increase of IFI16 was seen in U373 cells (**Figure 2B**).

### 3.3 Poly(dA:dT) induces IFNs and ISGs

**Figure 3:**
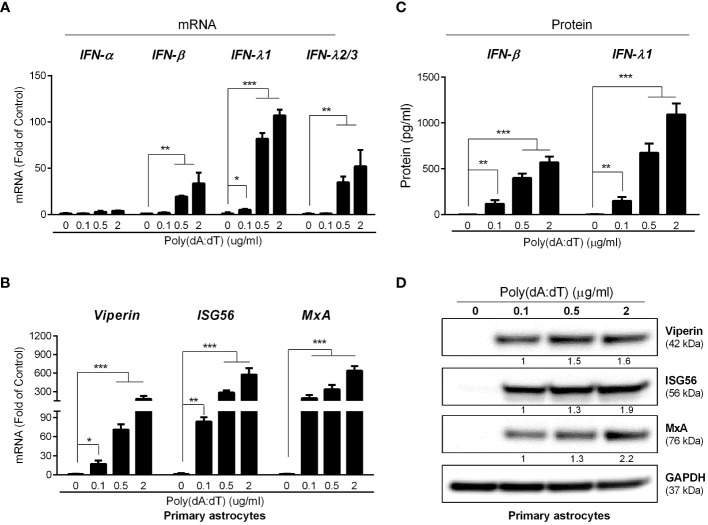
Poly(dA:dT) elicits IFNs and ISGs. The primary human astrocytes were treated with poly(dA:dT) at the indicated concentrations. After 12h, cellular RNAs were extracted and subjected to the real-time PCR for mRNA levels of IFNs **(A)** and ISGs **(B)**. **(C)** The cell-free supernatant was analyzed by ELISA to determine IFNs protein level. **(D)** The cellular proteins were extracted and subjected to Western blot for the indicated ISGs protein level. Protein expression relative to internal control is quantified using Image J software. Data shown in **(A–C)** were the mean ± SD from three independent experiments with triplicate wells (*P < 0.05, **P < 0.01, and ***P < 0.001).

We next determined the effect of poly(dA:dT) on IFN signaling pathway in primary human astrocytes. As shown in **Figures 3A, C**, poly(dA:dT) treatment of the cells resulted in significantly higher expression of IFN-β, IFN-λ1 and IFN-λ2/3 at both mRNA and protein levels. In addition, poly(dA:dT) treatment also dose-dependently induced the expression of the ISGs (MxA, ISG56 and Viperin) at both mRNA and protein levels (**Figures 3B, D**). Similarly, poly(dA:dT)-treated U373 cells had higher expression of IFNs and ISGs, and the effect of poly(dA:dT) was dose-dependent (**Supplementary Figure 1**).

### 3.4 Poly(dA:dT) induces IRFs

**Figure 4:**
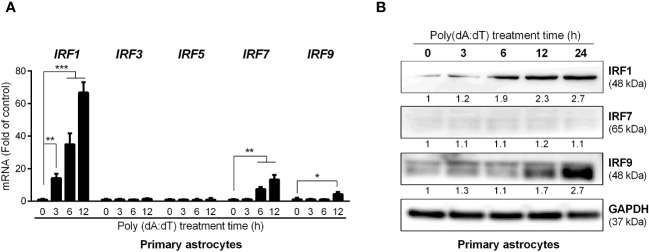
Poly(dA:dT) induces IRFs. Primary human astrocytes were treated with 0.5μg/ml of poly(dA:dT) for the indicated times. The total RNAs **(A)** and cellular proteins **(B)** were extracted for IRFs detection at both mRNA and protein levels. Protein expression relative to internal control is quantified using Image J software. Data shown in **(A)** were the mean ± SD from three independent experiments with triplicate wells (*P < 0.05, **P < 0.01, and ***P < 0.001).

Among nine members of human IRF family, IRFs 1, 3, 5, 7 and 9 are known to be involved in IFN-mediated immune regulation. We thus examined the effect of poly(dA:dT) on the expression of these five IRFs in human astrocytes. While poly(dA:dT) had little effect on IRF3 and IRF5 expression, it induced IRF1, IRF7 and IRF9 expression at both mRNA (**Figure 4A**) and protein levels (**Figure 4B**). Comparing with IRF7 and IRF9, IRF1 was induced at earlier time point (3h) and significantly higher level (**Figure 4**). Similar to primary human astrocytes, poly(dA:dT)-treated U373 cells also showed higher mRNA expression of IRF1, IRF7 and IRF9 (**Supplementary Figure 2A**). Increased IRF1 and IRF9 protein levels were also observed in U373 cells following poly(dA:dT) treatment, and IRF1 was induced earlier than IRF9 in U373 cells as well (**Supplementary Figure 2B**).

### 3.5 IRF1 knockdown diminishes poly(dA:dT)-induced ISGs

**Figure 5:**
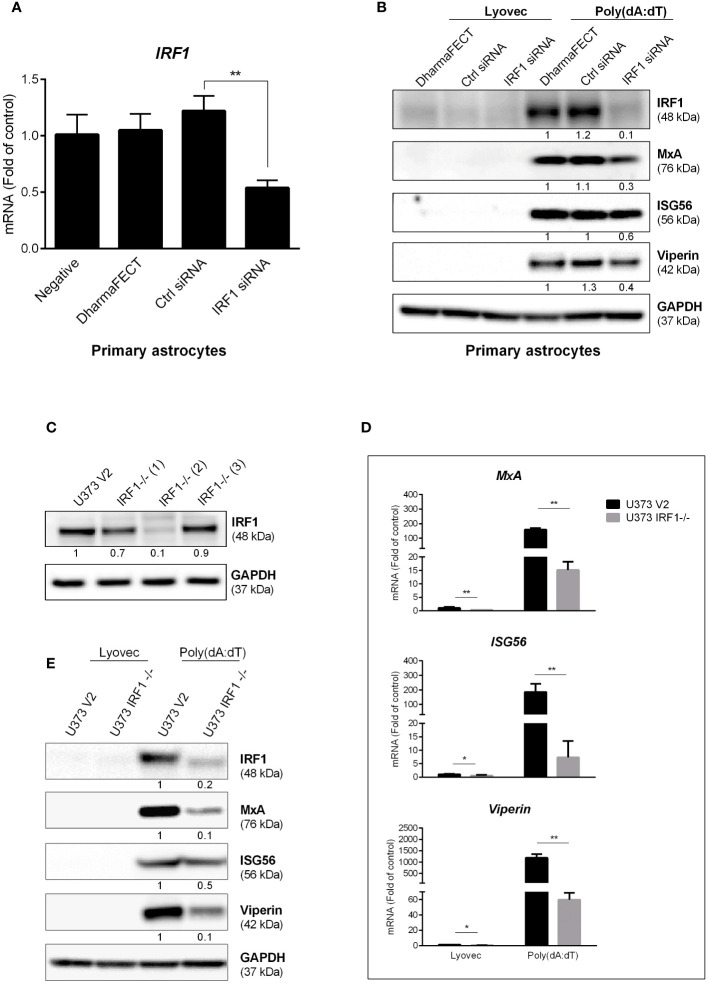
IRF1 knockdown inhibits poly(dA:dT)-induced ISGs. **(A)** Primary human astrocytes were transfected with 30nM of control siRNA or IRF1 siRNA for 24h. IRF1 mRNA level was determined by the real-time PCR. **(B)** Primary human astrocytes were transfected with 30nM of siRNA for 24h, and then treated with poly(dA:dT) for 24h. Proteins were extracted and subjected to Western blot. **(C)** CRISPR/Cas9 technology was used to reconstruct U373 V2 and U373 IRF1^-/-^ cells, the cellular IRF1 protein level was analyzed by Western blot. U373 V2 and U373 IRF1^-/-^ were treated with poly(dA:dT) for 12h or 24h, RNAs **(D)** and proteins **(E)** were collected and subjected to the real-time PCR or Western blot. Protein expression relative to internal control in **(B, C, E)** is quantified using Image J software. Data shown in **(A, D)** were the mean ± SD from three independent experiments with triplicate wells (*P < 0.05, **P < 0.01).

Based on the finding that poly(dA:dT) selectively induced some of IRF family members, particularly IRF1, we next studied whether IRF1 is a key regulatory factor in poly(dA:dT)-mediated the induction of the ISGs in primary human astrocytes. We observed that IRF1 knockdown by IRF1 siRNA resulted in the inhibition of poly(dA:dT)-induced ISGs expression in primary human astrocytes (**Figures 5A, B**). To further determine the role of IRF1 in poly(dA:dT)-mediated innate immunity, we constructed stable IRF1-knockout cell lines and demonstrated that one (U373 IRF1^-/-(2)^) of these lines expressed little IRF1 (**Figure 5C**). Using this cell line, we found that IRF1 knockout remarkably inhibited not only basal mRNA levels of MxA, ISG56 and Viperin (**Figure 5D**), but also poly(dA:dT)-induced expression of these ISGs at protein levels (**Figure 5E**).

### 3.6 IRF1 knockout suppresses poly(dA:dT)-induced IFNs and STATs

**Figure 6:**
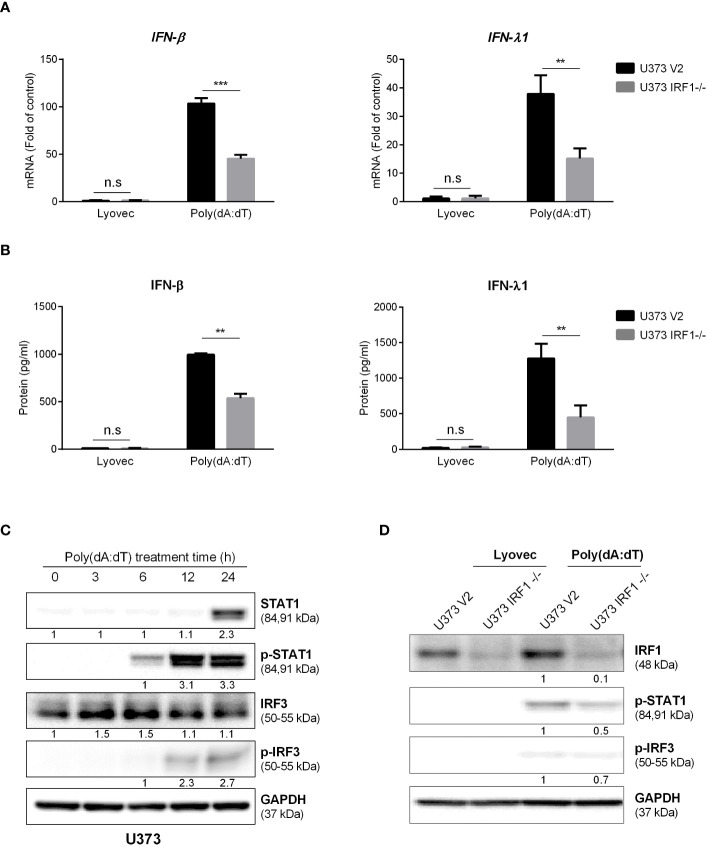
IRF1 knockout diminishes poly(dA:dT)-mediated activation of IFNs/STAT signaling. U373 V2 and U373 IRF1^-/-^ cells were treated with 0.5μg/ml poly(dA:dT) at indicated time points. Cellular RNAs **(A)** and cell-free supernatant **(B)** were then collected and subjected to the real-time PCR or ELISA for IFNs. **(C, D)** The cellular proteins were collected at indicated time points and subjected to Western blot for IRFs or STATs at the indicated times. Protein expression relative to internal control is quantified using Image J software. Data shown in **(A, B)** were the mean ± SD from three independent experiments with triplicate wells (**P < 0.01, ***P < 0.001). n.s: no significance.

In addition to the ISGs, we also examined the role of IRF1 in poly(dA:dT)-mediated IFN and STAT expression in astrocytes. As shown in **Figures 6A, B**, while there was little difference in the basal levels of IFNs between IRF1 knockout cell line and the control cells, inductive effect of poly(dA:dT) on IFN-β and IFN-λ expression at both mRNA and protein was attenuated in IRF1-knockout cell line as compared to the control cell line. In addition, we found that poly(dA:dT) time-dependently induced the expression of STAT1, p-STAT1 and p-IRF3 in U373 cells (**Figure 6C**). Interestingly, poly(dA:dT)-mediated p-STAT1 and p-IRF3 induction was diminished in U373 IRF1^-/-^ cells (**Figure 6D**).

### 3.7 IRF1 knockout inhibits IFNs-stimulated ISGs

**Figure 7:**
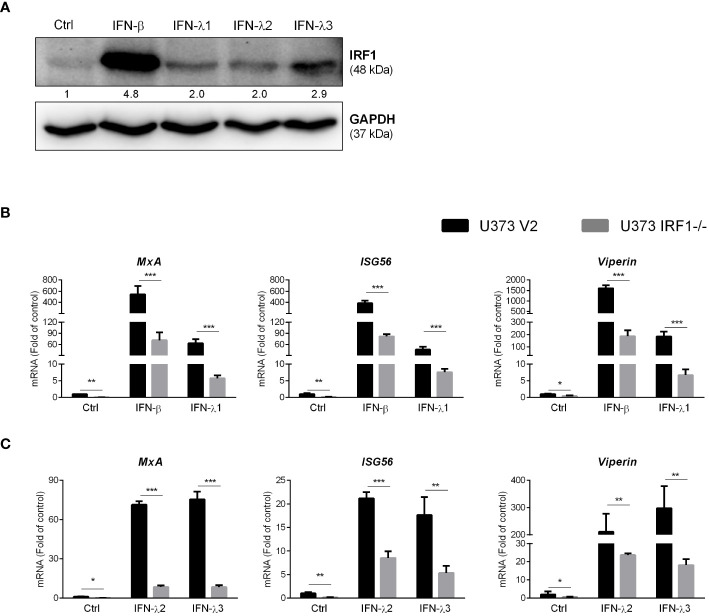
IRF1 knockout inhibits recombinant IFNs-stimulated ISGs. **(A)** U373 cells were treated with 100ng/ml IFN-β, IFN-λ1, IFN-λ2 and IFN-λ3 for 24h, the total proteins were collected to determine IRF1 expression. Protein expression relative to internal control is quantified using Image J software. **(B, C)** U373 V2 and U373 IRF1^-/-^ were treated with the recombinant IFN-β or IFN-λ1 or IFN-λ2 or IFN-λ3 for 12h. The total RNAs were extracted and subjected to the real-time PCR. Data shown in **(B, C)** were the mean ± SD from three independent experiments with triplicate wells (*P < 0.05, **P < 0.01, ***P < 0.001).

To determine the association of IFNs with IRF1 expression, we treated cells with or without the recombinant IFN-β- and IFN-λs. As shown in **Figure 7A**, the recombinant IFNs-treated U373 cells expressed higher levels of IRF1 as compared to the untreated cells. As compared to IFN-λs, IFN-β was more effective in IRF1 induction. We then investigated whether IRF1 knockout influenced recombinant IFNs-mediated ISGs expression. As demonstrated in **Figures 7B, C**, IRF1-knockout cells showed the reduced response to the recombinant IFNs in terms of the induction of MxA, ISG56 and Viperin.

### 3.8 The role of IRF1 in poly(dA:dT)-mediated HSV-1 inhibition in astrocytes

**Figure 8:**
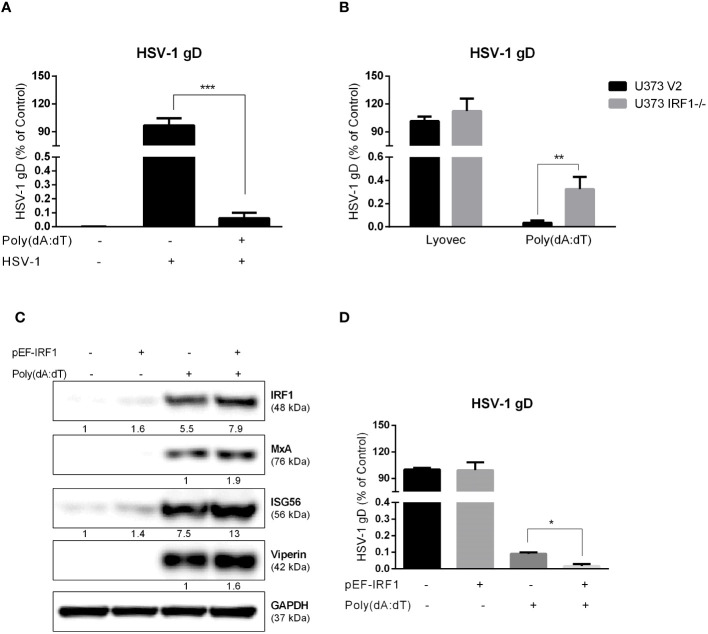
The role of IRF1 in HSV-1 inhibition by poly(dA:dT) in astrocytes. **(A)** U373 cells were treated with 0.1μg/ml poly(dA:dT) for 24h, and then infected with HSV-1 (MOI of 0.02) for 24h. **(B)** U373 V2 and U373 IRF1^-/-^ were treated with poly(dA:dT) for 24h, and then infected with HSV-1 for 24h. The cellular genomic DNAs were extracted and subjected to the real-time PCR using the specific HSV-1 gD primers for HSV-1 quantification. **(C)** U373 cells were treated with 1μg control vector or pEF-IRF1 for 24h, then stimulated with 0.1μg/ml poly(dA:dT) for 24h. Proteins were extracted and subjected to Western blot. Protein expression relative to internal control is quantified using Image J software. **(D)** pEF-IRF1 vector was transfected into U373 cells for 24h, stimulated with 0.1μg/ml poly(dA:dT) for 24h, then infected with HSV-1 (MOI of 0.02) for 24h. HSV-1 quantification was determined by the real-time PCR. Data shown in **(A, B, D)** were the mean ± SD from three independent experiments with triplicate wells. (*P < 0.05, **P < 0.01, ***P < 0.001).

We observed that poly(dA:dT) treatment could potently inhibit HSV-1 DNA replication in astrocytes (U373 V2) at 24h PI (**Figure 8A**). However, poly(dA:dT)-mediated suppression of HSV-1 gD gene expression was reduced in U373 IRF1^-/-^ cells (**Figure 8B**). We then examined the effect of IRF1 overexpression on poly(dA:dT)-induced ISGs expression and anti-HSV-1 effects. As shown in **Figure 8C**, IRF1 overexpression enhanced MxA, Viperin and ISG56 production induced by poly(dA:dT). In addition, IRF1 overexpression increases poly(dA:dT)-driven inhibition of HSV-1 gD DNA level (**Figure 8D**).

## 4 Discussion

The CNS innate immunity is crucial in determining the consequences of HSV-1 infection. Therefore, it is of a great interest to understand the role of intracellular innate immunity in protecting astrocytes from HSV-1 infection. We thus studied whether poly(dA:dT), a potent DNA sensors ligand, can activate intracellular immune response to HSV-1 infection/replication. We observed that comparing to untreated cells, poly(dA:dT)-treated astrocytes produced significantly higher levels of IFN-β and IFN-λ (**Figures 3A, C**), both of which can activate JAK-STAT pathways and induce the ISGs expression through a STAT1-STAT2-IRF9 heterotrimer (Rauch et al., 2013). Several *in vitro* studies highlighted the important role of type I IFNs in controlling the replication, spread, and cytopathic effect of HSV-1 infection (Sainz and Halford, 2002; Rosato and Leib, 2014). Like type I IFNs, type III IFNs also have a broad antiviral activity as they utilize the same signaling pathways as type I IFNs (Zanoni et al., 2017; Lazear et al., 2019). Li et al. reported that exogenous treatment of primary human astrocytes and neurons with IFN-λ inhibited HSV-1 gene expression and viral protein synthesis, through the induction of endogenous type I IFN production and ISG expression (Li et al., 2011). We demonstrated that poly(dA:dT) promoted astrocytes to produce several antiviral ISGs (Viperin, ISG56 and MxA) (**Figures 3B, D**). These ISGs are known to have the ability to inhibit virus replication at different levels (Fensterl and Sen, 2011; Fitzgerald, 2011; Bordi et al., 2013). For example, viperin could interact with HSV-1 gD protein and inhibit the viral proliferation (Li et al., 2019). ISG56 (Zhu et al., 2022) (Zhang R. et al., 2023) and MxA (Ku et al., 2011) (Tajpara et al., 2019) have been considered as classic antiviral factors involved in wild-type HSV-1 infection as well.

Studies have shown that PRRs including both DNA and RNA sensors (cGAS, DHX, IFI16, RIG-I, MDA5 and TLRs) are critical in controlling HSV-1 replication and dissemination (Alandijany, 2019). Several DNA sensors (IFI16, cGAS, and ZBP1) can detect intracellular viral DNA and activate the innate immunity (Veeranki and Choubey, 2012; Li et al., 2013; Kuriakose et al., 2016). We observed that poly(dA:dT) treatment could induce the expression of both DNA and RNA sensors in the astrocytes (**Figures 1**, **2**). Activation of these sensors can trigger IFN-JAK/STAT signaling pathways and induce the antiviral cellular factors. Suresh et al. showed that the DNA sensors (IFI16, ZBP1 and AIM2) were involved in poly(dA:dT)-induced the agonistic activation of cellular sensors (Suresh et al., 2021). We previously reported that poly(dA:dT) could exert anti-HSV effects in the epithelium systems primarily through RIG-I (Shao et al., 2020; Huang et al., 2022). Das et al. showed that two astrocyte cell lines displayed ZIKV-resistance when pretreated with poly(dA:dT), although the specific PRRs involved in the process were not identified (Das et al., 2022). Given the complexity of the interplays between the sensors, it is challenging to determine one specific DNA or RNA sensor that is responsible for the effects of poly(dA:dT) on HSV-1 and innate immune response in human astrocytes. However, it is still important to understand how the interplays take place between DNA and RNA sensors which contribute to host cell protection against HSV-1 infection.

The roles of IRF family members in innate immunity have been extensively studied. Given the key role of IRFs in regulating IFNs, we studied whether poly(dA:dT) has impact on IRF expression in astrocytes. We found that several IRFs, particularly IRF1, were significantly upregulated in poly(dA:dT)-treated astrocytes (**Figure 4**, **Supplementary Figure 2**). The role of IRF1 in the IFNs and ISGs induction by poly(dA:dT) was confirmed by the following observations: 1. IRF1 knockdown by IRF1 siRNA in primary astrocytes compromised poly(dA:dT)-induced ISGs expression (**Figures 5A, B**); 2. The inductive effect of poly(dA:dT) on the ISGs was attenuated in IRF1-knockout cell line (**Figures 5D, E**); 3. IRF1 knockout diminishes poly(dA:dT)-elicited IFNs expression (**Figures 6A, B**); 4. IRF1 knockout compromised poly(dA:dT)-induced the expression of p-IRF3 and p-STAT1 (**Figure 6D**); 5. The ability of the recombinant IFNs to stimulate ISGs expression was diminished in IRF1 knockout cell line (**Figure 7**). Comparing with IFN-λs, IFN-β induced the highest expression of IRF1, which is consistent with a previous report that IFN-β mediates early and transient IRF1 expression, whereas IFN-λ1 induces lower but continuous IRF1 expression (Zhou et al., 2022).

The IRF1 gene is highly responsive to a variety of stimuli, such as viruses, dsRNA, retinoic acid, IFNs and NF-κB, which have been investigated extensively (Feng et al., 2021). IRF1 is known to have momentous physiological significance in IFNs-induced positive feedback regulation. Among IRF family members, IRF1 was the first one identified to be involved in the transcription of IFN-β and other regulatory DNA elements (Fujita et al., 1988). While IRF3 and IRF7 have been considered as the mainstream regulatory factors in IFNs signaling pathway (Hiscott, 2007; Johnson et al., 2020), the function of IRF1 in antiviral innate immunity is recently becoming a focus of research (Feng et al., 2021). For instance, IRF1 in macrophages induces IFN-β and mount anti-Dengue virus responses even in the absence of IRFs 3, 5, and 7 (Carlin et al., 2017). Wang et al. (2020) showed that IRF1 could interact with IRF3 and block its interaction with protein phosphatase 2A to enhance the IRF3 phosphorylation, promoting the innate immune response to viral infection. Our data support the previous study (Panda et al., 2019) showing that IRF1 could induce early IFNs production by partially enhancing phosphorylation and localization of IRF3 without affecting the IRF3 transcription (**Figure 6C**). The role of IRF1 in DNA sensing-mediated IFNs-ISGs regulation was also evidenced in the experiments to examine the impact of poly(dA:dT) on STAT expression. We observed that while poly(dA:dT) could induce STAT1 expression at 24h post treatment, the increased phosphorylated STAT1 was found as early as 6h after poly(dA:dT) stimulation (**Figure 6C**), suggesting that facilitating STAT1 phosphorylation be the first step in the ISGs induction by poly(dA:dT). However, the phosphorylation of STAT1 induced by poly(dA:dT) was suppressed in IRF1 knockout cells (**Figure 6D**). In addition, using both IRF1 knockout and overexpression systems, we showed IRF1 was a critical regulator in restricting HSV-1 replication in human astrocytes (**Figures 8B–D**).

In summary, we have provided for the first time the experimental evidence that DNA sensor activation could trigger IFNs-JAK/STAT pathway through IRF1 and induce multiple intracellular HSV-1 restriction factors to inhibit the virus at different steps of its replication. Therefore, using DNA sensor ligands may represent a promising novel strategy for HSV-1 treatment. Because this strategy can activate the intracellular immunity with the production of multiple anti-HSV-1 factors in infected host cells, it is unlikely for HSV-1 to mutate and develop resistance. However, future *ex vivo* and *in vivo* investigations with animal models and clinical specimens are necessary, not only for confirming our *in vitro* findings, but also for developing DNA sensor activation-based immune therapy for HSV-1 infection of the CNS.

## Data availability statement

The original contributions presented in the study are included in the article/**Supplementary Material**. Further inquiries can be directed to the corresponding author.

## Author contributions

YL: Writing – original draft, Conceptualization, Data curation, Visualization, Writing – review & editing, Formal analysis, Investigation. X-QX: Methodology, Software, Writing – review & editing. W-JL: Methodology, Formal analysis, Writing – review & editing. BZ: Investigation, Writing – review & editing. F-ZM: Validation, Writing – review & editing, Investigation. XW: Project administration, Supervision, Writing – review & editing. SM: Formal Analysis, Writing – review & editing. ZG: Writing – review & editing, Investigation. W-ZH: Writing – review & editing, Project administration, Supervision, Writing – original draft, Conceptualization.
