# Supplementary material for: Cytosolic DNA sensors activation of human astrocytes inhibits herpes simplex virus through IRF1 induction
Source: Front Cell Infect Microbiol. 2024 May 14;14:1383811. doi: 10.3389/fcimb.2024.1383811 (PMC11130358; doi:10.3389/fcimb.2024.1383811)
Supplement: Supplementary file 1 [file DataSheet_1.pdf]

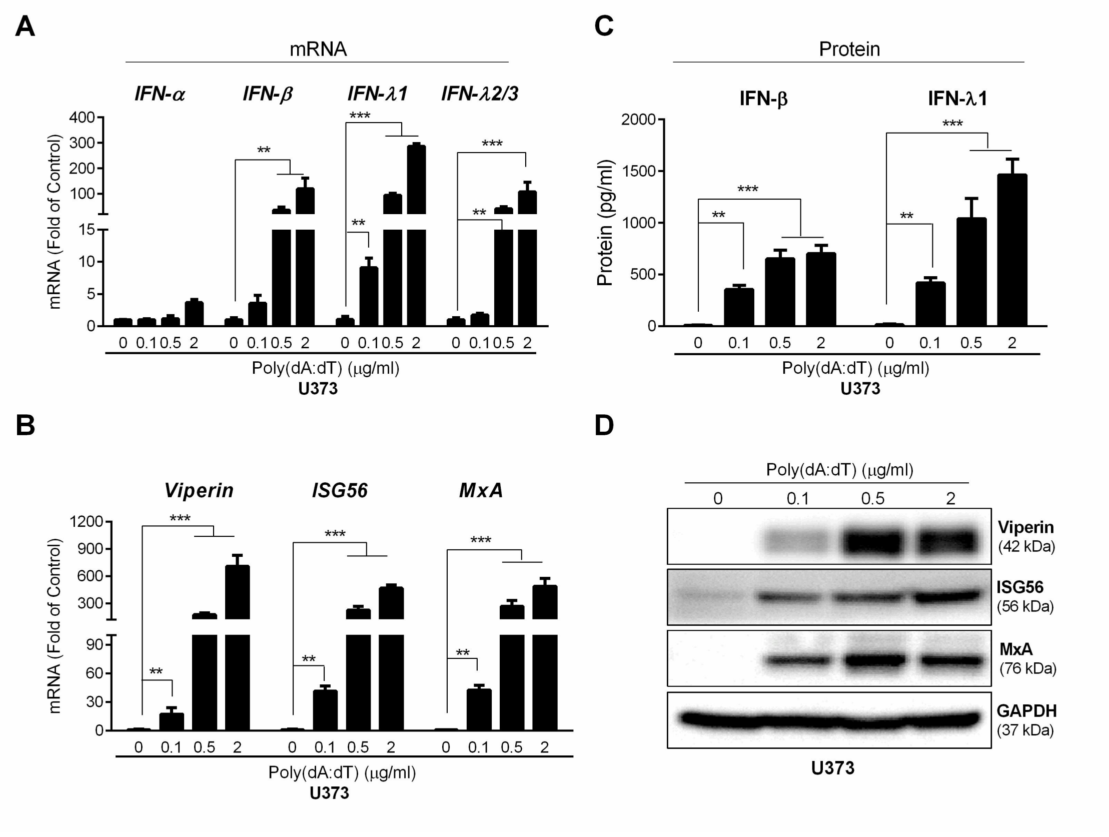

Supplemental Fig 1. Poly(dA:dT) induces IFNs and ISGs expression in U373 cells. Cellular RNAs were extracted at 12h after stimulation, and the mRNA levels of (**A**) IFNs and (**B**) ISGs were analyzed by real-time PCR. After 24h, (**C**) the cell-free supernatant was subjected to ELISA assay to determine IFNs expression, and (**D**) cellular proteins were subjected to Western blot to determine ISGs protein level. Data shown in Figs. A, B, and C were the mean ± SD from three independent experiments with triplicate wells (***P* < 0.01, ****P* < 0.001).

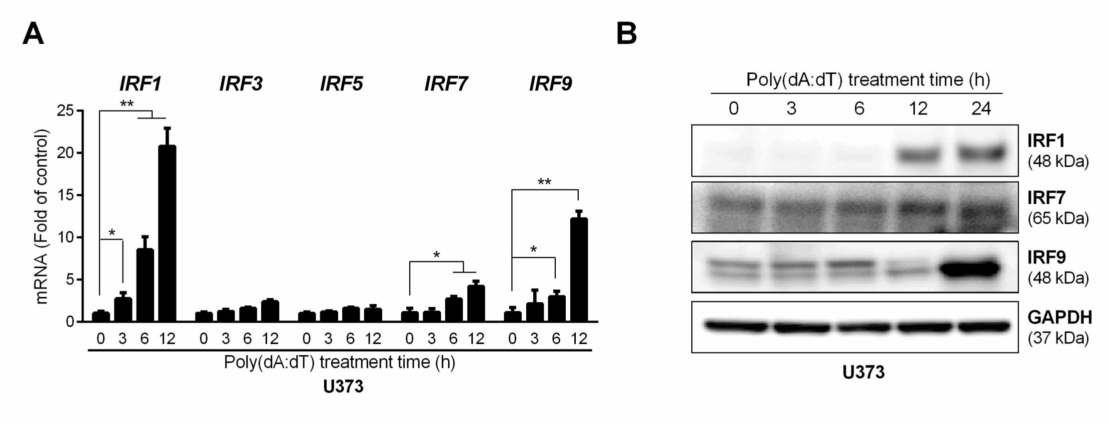

Supplemental Fig 2. Poly(dA:dT) induces IRF1 expression in U373. U373 cells were transfected with 0.5μg/ml poly(dA:dT) for the indicated times. The total RNAs (**A**) and cellular proteins (**B**) were extracted for IRFs detection. Data shown in Fig.A were the the mean ± SD from three independent experiments with triplicate wells (**P* < 0.05, ***P* < 0.01).
